# Factors influencing early introduction of complementary feeding in children under 2 years in Limpopo province

**DOI:** 10.4102/curationis.v48i1.2799

**Published:** 2025-10-17

**Authors:** Ndiambani A. Makhavhu, Ndidzulafhi S. Raliphaswa, Mphedziseni E. Rangwaneni

**Affiliations:** 1 Department of Advanced Nursing Science, Faculty of Health Sciences, University of Venda, Thohoyandou, South Africa

**Keywords:** children under 2 years, early complementary feeding, early introduction, factors, feeding practices, infant nutrition

## Abstract

**Background:**

An infant needs adequate, safe and appropriate complementary foods, with continued breastfeeding, at the age of six for survival, growth and development. However, young mothers stopped breastfeeding their children earlier than the recommended time and this often leads to the early introduction of food before the target age of 6 months. If this continues, malnutrition in children under 2 years will increase, leading to growth retardation, delayed mental development and impaired intellectual level.

**Objectives:**

This article aimed to determine factors influencing early introduction of complementary feeding in children under 2 years in the selected hospitals of Vhembe District, Limpopo province.

**Method:**

A qualitative approach was adopted. Purposive sampling was used to select four hospitals with high admission rates of malnutrition in the Vhembe District of the Limpopo province. Unstructured focus group discussions were conducted with 32 young mothers who were purposively sampled. Data were analysed using Tesch’s eight steps. Measures to ensure trustworthiness were employed. Ethical considerations were adhered to throughout the study.

**Results:**

The study findings revealed that young mothers had different knowledge about infant feeding of children under 2 years of age because of cultural beliefs and insufficient information on appropriate food types. This may have a negative impact on the normal growth and development of children as they lack proper nutrients.

**Conclusion:**

The study provided knowledge of varied beliefs and misconceptions regarding complementary feeding.

**Contribution:**

This study adds information to improve young mothers’ knowledge about complementary feeding.

## Introduction

Appropriate complementary feeding is critical in growth and development, especially in the first 2 years of life, which is the critical window of opportunity. As an infant develops and becomes more active, particularly from the age of 6 months, breast milk falls short of the infant’s nutritional requirements. This gap continues to widen as babies and young children get older. To close these gaps, complementary feeding is crucial and should be timely, adequate and safe (World Health Organization [WHO] 2021). Complementary feeding refers to an important core indicator of infant and young child feeding practices and the transition involving the introduction of solid and semisolid foods to the infant’s diet when realising that breast milk alone is no longer sufficient to meet the nutritional requirements of an infant (WHO 2021). Furthermore, WHO recommended that mothers worldwide should exclusively breastfeed their infants for the first 6 months and thereafter gradually receive complementary foods that are nutritionally adequate and safe until 2 years old or older. Complementary feeding should be given between the ages of 6 and 23 months with continued breastfeeding, when most infants reach a general and neurological stage of development that enables them to be fed other foods rather than breast milk (Harish & Madhu 2018). This period is regarded as the most ‘critical window’ for the promotion of health and proper growth and development of children and is the period when children’s status gets compromised. Appropriate complementary feeding plays an essential role in preventing malnutrition in children under the age of 2 years (United Nations Children’s Fund [UNICEF] 2021). In Limpopo province, 25.9% of children died as a result of severe acute malnutrition (SAM) (Gavhi et al. 2020).

Early introduction of complementary feeds before the age of 6 months can lead to the displacement of breast milk and increased risk of infections such as diarrhoea, which further contribute to weight loss and malnutrition because of immaturity of the kidneys, gastrointestinal and neurodevelopmental systems (Kandel et al. 2016). While it is estimated that only 44% of infants worldwide are exclusively breastfed for the first 6 months of life, there is an opportunity for improvement in infant feeding practices. Many infants are being introduced to solid, semi-solid, or soft foods earlier than recommended, highlighting the need for increased awareness and education about proper infant nutrition (Muleka et al. 2023). South Africa (SA) is one of the African regions with the lowest rates of breastfeeding, where only 7.4% of children were exclusively breastfed for the first 6 months, while most of the children received solid food and fluids earlier than the recommended age of 6 months (UNICEF 2021). There is an opportunity to improve infant nutrition, as currently less than 1% of children are exclusively breastfed for up to 3 months in the Limpopo province, Dzimauli region. Encouraging and supporting breastfeeding practices could significantly enhance the health and well-being of infants in this community. However, none of the children were breastfed exclusively up to the age of 6 months (Mushaphi et al. 2017). Several studies conducted in SA found that complementary food is introduced to children before the target age of 6 months as per the WHO recommendations (Chakona 2020; Mulenga, Amukugo & Shilunga 2018). Poor breastfeeding and complementary feeding practices account for illness, infections such as diarrhoea and nutrient deficiency in children that lead to malnutrition at an early age (Molla, Ejigu & Nega 2017). In a recent study conducted in Limpopo province, 25.9% of children died as a result of SAM (Gavhi et al. 2020). Inappropriate feeding practices, such as mixed feeding and early introduction of solids, semi-solids and soft foods, were identified as major contributors to child malnutrition in Limpopo province (Mugware, Motadi & Mushaphi 2022). Researchers found that cultural beliefs and insufficient information of mothers and caregivers were the major contributory factors of poor complementary feeding (Cascone et al. 2019; Cook et al. 2020; Karmee, Satapathy & Tripathy 2018; Mohammed et al. 2018). In most cases, young mothers lack information related to exclusive breastfeeding, breastfeeding duration, when to start complementary feeding and different types of complementary foods for children under 2 years (Mulenga 2018). Because of a lack of information on complementary feeding, young mothers stop breastfeeding their children early, leading to the introduction of complementary food before 6 months of age, which contributes to malnutrition. Despite the information, policies, preventative strategies and guidelines that are accessible and available, children under the age of 2 years of young mothers are still hospitalised with malnutrition in Limpopo province. Hence, this study seeks to determine factors influencing the early introduction of complementary feeding in children under 2 years in Limpopo province.

## Materials and methods

### Research design

A qualitative research approach was adopted using an explorative, descriptive and contextual research design. The approach was suitable in this study because young mothers described their experiences regarding complementary feeding practices in children under 2 years.

### Study setting

The research was carried out in four hospitals within the Vhembe District, which is one of the five districts in Limpopo province. These facilities were specifically chosen from a total of seven hospitals based on their notably high rates of malnutrition-related admissions among children.

### Study population

This study focused on young mothers aged 18–26 years who had children under 2 years old admitted to the Paediatric Medical Ward (PMW) for malnutrition. The selection of this age group was intentional, as they were identified as experiencing notable challenges related to childhood malnutrition. Investigating this specific population was therefore essential to explore potential underlying causes.

### Sampling method

A purposive sampling technique was employed, targeting young mothers aged 18–26 years whose children under the age of two were admitted to PMWs for malnutrition. This group was selected because of their direct experience with complementary feeding practices for infants. Among the five districts in Limpopo province, Vhembe District was specifically chosen owing to its notably high mortality rates associated with child malnutrition. Furthermore, four hospitals were purposively selected from the seven facilities in the district that reported the highest admission rates of malnourished children under 2 years.

### Sample size

The study included a total of 32 participants, organised into six focus group discussions (FGDs). Two FGDs were conducted at a referral hospital, with one focus group comprising six young mothers and the other group five young mothers. The remaining four FGDs were held across three selected hospitals, involving groups of 11 (2 FGDs each with 11 participants), five and six participants, respectively. Data saturation was reached by the fourth focus group; however, the researcher proceeded with two additional groups to confirm that no new themes or information were emerging.

### Data collection

The PMWs of the selected hospitals were visited after permission had been granted, and permission was sought from the operational manager (OM). After permission was granted by OM, the researcher asked for the admission register book to check all children under 2 years admitted with malnutrition only. All young mothers were recruited to participate in the study who met the inclusion criteria. A consent form to sign was given to young mothers, who were given a clear explanation that they were the relevant sources of information for the study.

Unstructured interviews through FGDs with young mothers of children admitted with malnutrition in PMWs were used to collect data. Non-verbal cues were also captured as participants were describing their lived experiences. Prior to the interview, participants were given consent forms to read and sign, and only verbal consent was given by participants who could not read or write or who preferred not to sign. Each interview ranged from approximately 60 min to 90 min. The open-ended interview was guided by one central question: ‘I would love to hear about the kinds of food you offer your children. It could really help to share ideas and inspire healthier meal choices!’ Different effective communication skills were used to get clear information from the participants, such as probing and active listening. Unstructured interviews were used because they focused on personal lived experiences. Interviews were audio recorded with the permission of participants throughout the duration of the interview. The findings of the study were shared and discussed collaboratively with the participants, providing an opportunity for valuable feedback and insights. Data were also transcribed verbatim. Measures to prevent COVID-19 were adhered to throughout the study during FGDs.

### Data analysis

Using Tesch’s open coding method as outlined by Creswell 2016, the data were carefully examined line by line to identify meaningful segments, which were then labelled with emerging concepts reflecting the participants’ responses. This inductive process facilitated the development of initial categories grounded in the data.

### Measures to ensure trustworthiness

To establish trustworthiness in this qualitative research, the study adhered to four core criteria: credibility, transferability, dependability and confirmability. Credibility was achieved through prolonged engagement with participants, peer debriefing and member checking to validate findings. Transferability was supported by providing rich, detailed descriptions of the research context and participants to allow others to assess the relevance of the findings to different settings. Dependability was ensured by maintaining an audit trail that documented the research process, while confirmability was strengthened through reflexive journaling and triangulation of data sources to minimise researcher bias.

### Ethical considerations

Prior to starting the research process, permission to conduct the study was obtained from the University of Venda Research Ethics Committee, which granted clearance certificate number (SHS/20/PDC/56/2701), the Department of Health Research Ethics Committee, Limpopo province, Vhembe District as well as four sampled hospitals. Participants were provided with the information regarding the study. Young mothers were informed that participation is voluntary to ensure no harm. Participants can withdraw at any moment without punishment being used against them. In this study, only codes were used. The information provided by the young mothers during the interviews was kept in a safe place.

## Results

This study comprised 32 young mothers: 11 from hospital one, 11 from hospital two, 5 from hospital three and 5 from hospital four. One theme emerged from the analysed data, namely, knowledge about infant feeding, and two subthemes were cultural beliefs and insufficient information on types of appropriate food.

### Theme: Knowledge about infant feeding

The study’s findings revealed that young mothers had different knowledge about infant feeding of children under 2 years of age, namely, cultural beliefs and insufficient information on types of appropriate food, and each is discussed next.

#### Cultural beliefs

From the analysed data, young mothers’ perceived cultural beliefs were found as one of the factors influencing complementary feeding. Cultural beliefs have a negative influence on young mothers’ recommended complementary feeding practices. This is illustrated in the following expression:

‘… I started to give food to my child when she turned five months old because I did not have enough breast milk …’ (Participant 2, Female, Focus Group 3)

‘… I don’t remember because my mother-in-law was the one who started giving her complementary food but it was before six months. I think it was when she was three months old after we visited her traditional healer. She said soft porridge mixed with some herbs is good for her and will grow well [*fidgeting her clothes*] …’ (Participant 1, Female, Focus Group 5)

‘… I was also told by my mother, to start giving her food because she was very small. She said if we start giving her food early, maybe she will gain weight very fast …’ (Participant 5, Female, Focus Group 4)

‘… My granny said to me that I must give her food because she was crying a lot due to hunger, and she also said that food would make her fat and healthy. [*looking down*] …’ (Participant 3, Female, Focus Group 1)

‘… I breastfed my child for up to four months because I was sick, and my breasts were painful. My mother told me to stop breastfeeding the baby because she will also end up ill …’ (Participant 4, Female, Focus Group 4)

#### Insufficient information on types of appropriate food

Participants in this study have insufficient experience with complementary feeding, as they do not know the types of appropriate food for children under 2 years. Young mothers attributed their insufficient information to the fact that they were not given any information related to complementary feeding for children under 2 years of age:

‘I was not told about the types of food by anyone, I tried to search on the internet on my own …’ (Participant 4, Female, Focus Group 1)

‘… Nobody told me when I must start feeding my child; that’s why I started giving her food at three weeks …’ (Participant 4, Female, Focus Group 5)

‘… I know nothing. I exclusively breastfed my child up to three months, then started giving her juice for children …’ (Participant 5, Female, Focus Group 3)

‘If I knew the correct food for my child, I wouldn’t feed her Mageu …’ (Participant 3, Female, Focus Group 1)

‘I don’t have any information about the correct food for my child. I don’t know what and when to give to my child. If I had information, my child would not have been admitted to this ward with Kwashiorkor. I would have given her nutritious food. [*crying*] …’ (Participant 4, Female, Focus Group 4)

## Discussion

It is evident from the study that insufficient information related to complementary feeding, as well as cultural beliefs, has contributed to the early introduction of complementary food in children under 2 years. Many young mothers tend to stop breastfeeding earlier than recommended, which can prompt the need for introducing complementary foods at an earlier stage. Encouraging education and support for extended breastfeeding could help improve the timing of complementary food introductions, as pointed out by other researchers (Mulenga et al. 2018; Tsega et al. 2021). In this study most young mothers feed their children under 2 years inappropriate foods such as danone, mageu, simba and rooibos tea for children because of a lack of information on complementary feeding. These findings are similar to those of a study conducted by Mugware et al. (2022), where children before the age of 6 months were given soft maize porridge mixed with water only as breakfast, lunch and supper throughout the week. In the Oshikoto region of Namibia, a lack of proper information on infant and child nutrition leads mothers and caregivers not to feed their children different types of food they produce, such as beans, pumpkins, wild spinach, groundnuts and fruits such as wild berries, palm fruits, fig fruits and marula fruits (Maciel 2017; Mulenga 2018). The most common complementary foods given to infants less than 6 months in Cape Town were cereals, yellow vegetables, yoghurt, porridge, chips, fruit juices and sweet beverages (Ikobah et al. 2023). It was also evident that young mothers who participated in this study perceived cultural beliefs as one factor influencing complementary feeding of children under 2 years. In SA, young mothers give newborn babies boiled water and herbs locally known as ‘isikhathi’ for colic (Marvin-Dowle, Soltani & Spencer 2021), these authors also indicated that culturally, young mothers in SA are supposed to practise abstinence from sexual intercourse with their partners for at least 3 months when they are breastfeeding to avoid impure breast milk, and this will lead to child sickness. In Limpopo province, in one region of Vhembe, babies were given traditional fermented soft porridge with different medicinal roots as the first food item to be introduced (Mbhenyane et al. 2023). This was because of a lack of adequate knowledge regarding complementary feeding because this study revealed that young mothers were not given information related to CF for children under 2 years old. Inadequate knowledge resulted in stopping breastfeeding early, initiation of CF early and feeding children with less frequency, leading to the development of malnutrition in their children. Despite the information in the Road to Health Booklets and the health education provided by health care professionals, in Limpopo province, complementary feeding is still a challenge. The World Health Organization/United Nations Children’s Fund recommended that to ensure that the child obtains all the required nutrients, complementary foods should be introduced at 6 months of age, and in this study, most young mothers were unable to do this (WHO & UNICEF 2023). Similarly, several studies conducted in South Africa reported the introduction of complementary foods before the recommended period of 6 months of age, which led to malnutrition (Mphasha et al. 2023; Mugwera et al. 2022; Mulenga et al. 2018). The mortality rate of children under 2 years of age can be prevented by appropriate breastfeeding and complementary feeding practices. It is therefore important for health care professionals to provide information regarding complementary feeding to young mothers to prevent malnutrition in children under 2 years.

### Strengths and weaknesses of the study

This study adds information to improve young mothers’ knowledge about complementary feeding. The limitation of the study is that the study did not cover the whole of Limpopo, as the study was only conducted in one district, and results cannot be generalised. The study is also limited to young mothers only, whereas it is a general problem that affects mothers at any age.

## Conclusion

The study findings highlight a critical need for increased education on complementary feeding for young mothers. Providing them with timely and accurate information could empower them to continue breastfeeding for the recommended duration and to introduce complementary foods at the appropriate time. Insufficient information related to complementary feeding and cultural beliefs can impact the nutritional status of a child, resulting in an irreversible outcome of malnutrition. Therefore, it is essential for health care professionals to disseminate information on complementary feeding to young mothers through all possible platforms, like social gatherings, to prevent malnutrition in children under the age of 2 years. Further studies are necessary to address misconceptions related to complementary feeding and early introduction of complementary foods.
